# Grappling with Chronic Pain and Poverty during the COVID-19 Pandemic

**DOI:** 10.1080/24740527.2020.1766855

**Published:** 2020-06-08

**Authors:** Fiona Webster, Laura Connoy, Abhimanyu Sud, Andrew D. Pinto, Joel Katz

**Affiliations:** Arthur and Sonia Labatt Family School of Nursing, Faculty of Health Sciences, Western University, London, Ontario, Canada; Department of Family and Community Medicine, Faculty of Medicine, University of Toronto, Toronto, Ontario, Canada; Upstream Lab, MAP/Centre for Urban Health Solutions, Li Ka Shing Knowledge Institute, Unity Health Toronto, Toronto, Ontario, Canada; Department of Family and Community Medicine, St. Michael’s Hospital, Toronto, Ontario, Canada; Institute for Health Policy, Management and Evaluation & Division of Clinical Public Health, Dalla Lana School of Public Health, University of Toronto, Toronto, Ontario, Canada; Department of Psychology, York University, Toronto, Ontario, Canada; Department of Anesthesia and Pain Management, Toronto General Hospital, Toronto, Ontario, Canada; Department of Anesthesiology and Pain Medicine, Faculty of Medicine, University of Toronto, Toronto, Ontario, Canada

The world is facing unprecedented challenges with the COVID-19 pandemic. This pandemic has drawn attention to the importance, failings, and weaknesses of health systems around the world, and brought health disparities into sharp relief.^1,2^ Governments and health-care systems are scrambling to find ways to minimize the downstream impacts of the pandemic on its citizens. Nevertheless, within Canada, as in many other countries, systemically and structurally marginalized populations are disproportionately susceptible to both COVID-19 infection and related morbidity and mortality. This includes those living in poverty^3^ and with chronic pain.^4,5^ The association between wealth and health has been well established for decades.^6,7^ In terms of this pandemic, those most at risk of dying if infected with COVID-19 are those with underlying chronic health conditions, including older people who have higher rates of persistent pain.^8^ And those who are poor are many times more likely to have multiple chronic conditions.^9^ This association has become urgently highlighted in recent weeks and includes people in prison,^10^ refugee populations,^11^ migrant workers,^12,13^ people sleeping rough,^2^ and people who use drugs.^14^ The neoliberal policies that effectively concentrate wealth amongst the few and result in poverty for others also reinforce notions that these members of society are less worthy of care.

In this editorial, we focus our attention on the implications of the COVID-19 pandemic for people with chronic pain, and in particular, those also living with low socioeconomic status (SES). Recent Canadian studies indicate that people with chronic illness including pain are much more likely to have low SES.^15^ For example, a clinic-based study from British Columbia identified a poverty rate of 50 percent among chronic pain patients.^16^ Such disparities are rooted in the social determinants of health – poverty, living and working conditions, social exclusion, etc. – within existing inequitable arrangements of oppression and domination. Existing biomedical conceptualizations of pain often construct pain in dualistic and reductionist ways, and thereby problematize people living with pain. People living with chronic pain are being disproportionately impacted by the recent rapid and drastic diversion of resources brought about by society’s response to COVID-19. The health system focus on preserving high acuity medical care has increased demands on community-based care which have not been sufficiently prepared or resourced to meet this challenge. The vast majority of chronic pain care is delivered in such community-based settings. As noted by Eccleston et al.^4^ these and other populations “with higher existing pain burden are more likely to experience higher incidence of COVID-19 infections, greater disruption to their usual healthcare access, and worse downstream consequences of abruptly altered health care.”

People living with pain also frequently experience co-morbidities such as addiction and mental illness which are now being unwittingly exacerbated by government responses to the coronavirus pandemic. Preliminary findings from our ongoing ethnographic research suggest that patients with chronic pain often feel stigmatized and misunderstood by care providers. This aligns with our interviews with physicians who described feeling overwhelmed by patients with chronic pain and were, at times, judgmental of their patients’ situations.^17^ Further, patients describe having to negotiate between different systems that are not coordinated, carry different understandings of the patient’s “primary problem,” and thus offer different solutions that are frequently incompatible across domains. This fragmentation of the care they receive occurs within the medical realm (that is, between mental health care and primary care for example) and between medical care and social services. Physical distancing measures and other social and economic restrictions have in many cases transformed these preexisting fragmentations into wide ruptures.

The health-care needs of this group already required greater attention prior to the emergence of COVID-19, particularly in relation to opioid use, yet these concerns have been effectively sidelined during this pandemic. Lifetime prevalence rates of chronic pain range from 16 to 45.4% depending on the population^18-20^ and increased rates of opioid prescribing have been linked to the high prevalence of chronic pain, alongside increases in opioid-related deaths.^21,22^ The College of Physicians and Surgeons of Ontario recently stated that Canada was experiencing “escalating overdose deaths in multiple provinces and the second highest rate of opioid prescribing/use per capita in the world^23^ [College, n.d.].” Very recent data show that 1 in 133 deaths in Ontario are opioid-related 22[Gomes et al., 2018]. While these alarming statistics no longer make headlines, some of the COVID-19 response measures are creating a barrier to accessing services for those living with pain, like pain management services^24^ and supervised consumption sites^25^ which is leading to a spike in overdoses.^26,27^ There have been some initial measures, such as Health Canada’s exemptions to the *Controlled Drugs and Substances Act* to allow for pharmacist extensions of controlled drug prescriptions and to allow physicians to issue verbal orders for controlled drugs, to accommodate the needs of people living in pain during the pandemic. Likewise, individual health-care providers, health organizations, and civil society organizations have rallied to collate virtual resources for people living with pain.

Although studies are emerging to address how chronic pain is being managed during the pandemic^4,5^ these do not yet include the voices of patients, and historically have not included the perspectives of those persons living with low SES.^28^ Tragically, many people have died as a result of COVID-19 infection, but we cannot forget that many have already lost their lives to overdose (4,623 Canadians died from opioids in 2018 alone) and this has risen during the time of the ongoing pandemic.^26^ We ask, as have our colleagues, in relation to other chronic conditions,^29^ how people living with chronic pain and also managing low or lost income and mental illness due to the COVID-19 pandemic are able to access virtual health care or much needed medications or support services? How do policies aimed at social distancing for example, affect those with inadequate housing or already struggling with isolation and living in pain?

We urgently need to understand how the pandemic and responses to it impact the health and lives of people living with chronic pain and low SES in ways both intended and unintended. Chronic pain and its associated morbidities such as mental illness and addiction cannot be understood through a strictly biomedical lens: consideration also must be given to the complex and multi-faceted nature of the social context.^16^ This adds a layer of additional stress to lives already marked by complexity and instability. Policies must intersect across social and health-care sectors, before, during and after this pandemic. Failing to do so can only lead to more harm for already marginalized groups.

It is our hope that by seeking to understand how the present COVID-19 pandemic affects the vulnerable population of people living with chronic pain and low SES, we will be able to strengthen and better integrate our public health, health care, and social care systems now and into the future and avoid reproducing the types of epistemic injustice that underpin and reproduce this group’s vulnerability and invisibility.^30^ Further, a focus on chronic pain may serve to highlight the experiences of people living with other complex chronic health conditions, who also require specialized help during this pandemic.
